# Biomimetic Restoration Through Fragment Re-attachment in Pediatric Dental Trauma: A Case Report

**DOI:** 10.7759/cureus.111884

**Published:** 2026-07-01

**Authors:** Sandisha S Sudrik, N.D. Shashikiran, Namrata Gaonkar, Ruchira R Sawant, Rutuja B Salunkhe

**Affiliations:** 1 Department of Paediatric and Preventive Dentistry, School of Dental Sciences, Krishna Vishwa Vidyapeeth, Karad, IND

**Keywords:** biomimetic restoration, crown fracture, dental trauma, fragment re‑attachment, pediatric dentistry

## Abstract

Dental trauma is one of the most prevalent problems seen in clinical practice. Crown fractures of anterior teeth are particularly common among children and adolescents. Such injuries have significant physical, psychological, and social impacts, necessitating immediate intervention.

This case report describes the management of an uncomplicated crown fracture in an eight‑year‑old girl following traumatic dental injury. The fractured fragment was retrieved, stored appropriately, and re‑attached using adhesive protocols. The procedure involved cleaning and conditioning of both the tooth and fragment, application of bonding agent, and re‑attachment with resin composite. Finishing and polishing restored esthetics and function.

Follow‑up was done after one week, followed by one month, and then at three months, revealing satisfactory esthetics, intact fragment retention, and absence of sensitivity or pulpal pathology. The patient and parents expressed high satisfaction with the conservative approach.

Fragment re‑attachment represents a biomimetic and conservative restorative option for managing traumatic crown fractures in pediatric patients. Advances in adhesive dentistry have made this technique reliable, predictable, and esthetically superior, preserving the patient’s own tooth tissue while minimizing chairside time and psychological distress.

## Introduction

Approximately 5% of all physical injuries involve the oral cavity, with traumatic dental injuries (TDIs) accounting for nearly 92% of patients seeking treatment for oral injuries [1]. Recognizing their high prevalence and impact, the WHO considers dental trauma a significant public health concern. The reported prevalence of TDIs ranges from 7.4% to 58% across different populations [2,3]. Among these injuries, anterior crown fractures are particularly common in children and adolescents, with a reported prevalence of approximately 18.8%. The prominent position and eruption pattern of the maxillary incisors make them especially susceptible to trauma resulting from falls, contact sports, road traffic accidents, and outdoor recreational activities [4].

TDIs can adversely affect a child's quality of life by impairing function, esthetics, speech, and psychosocial well-being, necessitating timely diagnosis and management. According to Andreasen's classification, crown fractures are broadly categorized as uncomplicated (involving enamel or enamel and dentin without pulp exposure) and complicated (with pulp exposure). The choice of treatment depends on several factors, including the type and extent of fracture, the patient's age, the stage of root development, and the condition of the fractured fragment [5]. Conventional treatment modalities include direct composite restorations, post-and-core build-ups, and full-coverage crowns. Although effective, these approaches often require additional removal of sound tooth structure and may not completely reproduce the esthetics and optical characteristics of the natural tooth.

With advances in adhesive dentistry, fragment re-attachment has evolved from being regarded as an interim procedure to a well-established biomimetic treatment option for the management of crown fractures [6-8]. The technique was first reported by Eidelman and Chosack in 1964 [9]. Subsequently, Simonsen described the circumferential enamel bevel technique in 1979, recommending a 45° bevel around both the fractured tooth and the fragment to improve adhesion and camouflage the fracture line. In 1982, he introduced the V-shaped internal enamel groove technique as a modification to enhance esthetics and overcome the potential discoloration associated with the labial composite band [10]. The International Association of Dental Traumatology (IADT) recommends fragment re-attachment whenever the fractured fragment is available, intact, and has been appropriately preserved [3].

Fragment re-attachment offers several advantages, including preservation of natural tooth structure, excellent esthetics through maintenance of the original enamel color, translucency, and surface texture, minimal chairside time, and significant psychological benefits for the child. Improvements in adhesive systems, restorative materials, and preparation designs have further enhanced the predictability and long-term success of this conservative treatment approach.

The present case report describes the successful management of an Andreasen Class II (uncomplicated crown fracture involving enamel and dentin without pulp exposure) in an eight-year-old girl using fragment re-attachment. The report highlights the importance of appropriate adhesive protocols and conservative treatment in achieving favorable esthetic and functional outcomes.

## Case presentation

**Figure 1:**
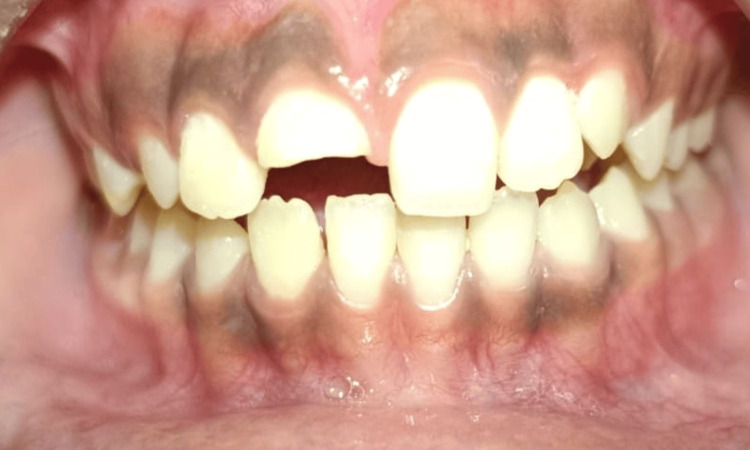
Pre‑operative intraoral photograph showing uncomplicated crown fracture of the maxillary central incisor (tooth #11) in an eight‑year‑old girl following traumatic dental injury.

**Figure 2:**
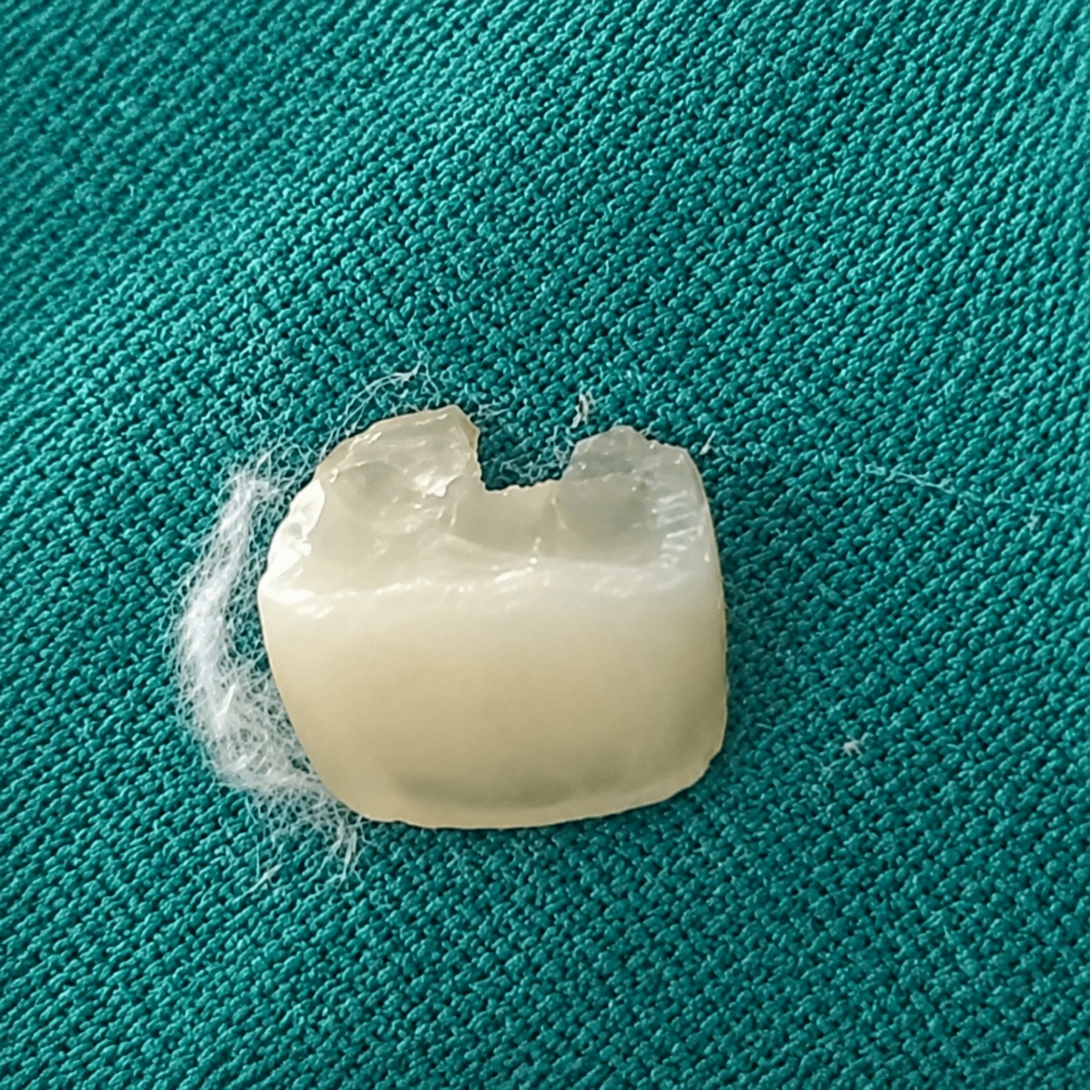
Fractured fragment of the maxillary right central incisor (tooth #11) retrieved immediately after trauma and stored in saline prior to re‑attachment.

An eight‑year‑old girl presented to the department of paediatric and preventive dentistry with a chief complaint of a broken upper front tooth following a fall while playing at school (Figure 1). The patient was otherwise healthy, with no relevant medical or dental history. Extraoral examination revealed no soft tissue injuries, while intraoral examination showed an uncomplicated crown fracture of the maxillary right central incisor (tooth #11). The fractured fragment had been retrieved by the parents immediately after the injury and was presented in good condition, without discoloration or contamination (Figure 2). The tooth was asymptomatic, with no mobility or tenderness to percussion.

A periapical radiograph confirmed the absence of pulpal involvement, root fracture, or periapical pathology, and the periodontal ligament space appeared normal. Based on clinical and radiographic findings, the case was diagnosed as an uncomplicated crown fracture of tooth #11. Considering the availability and intact condition of the fractured fragment, a conservative approach of fragment re‑attachment was planned. The procedure was explained to the patient and her parents.

**Figure 3:**
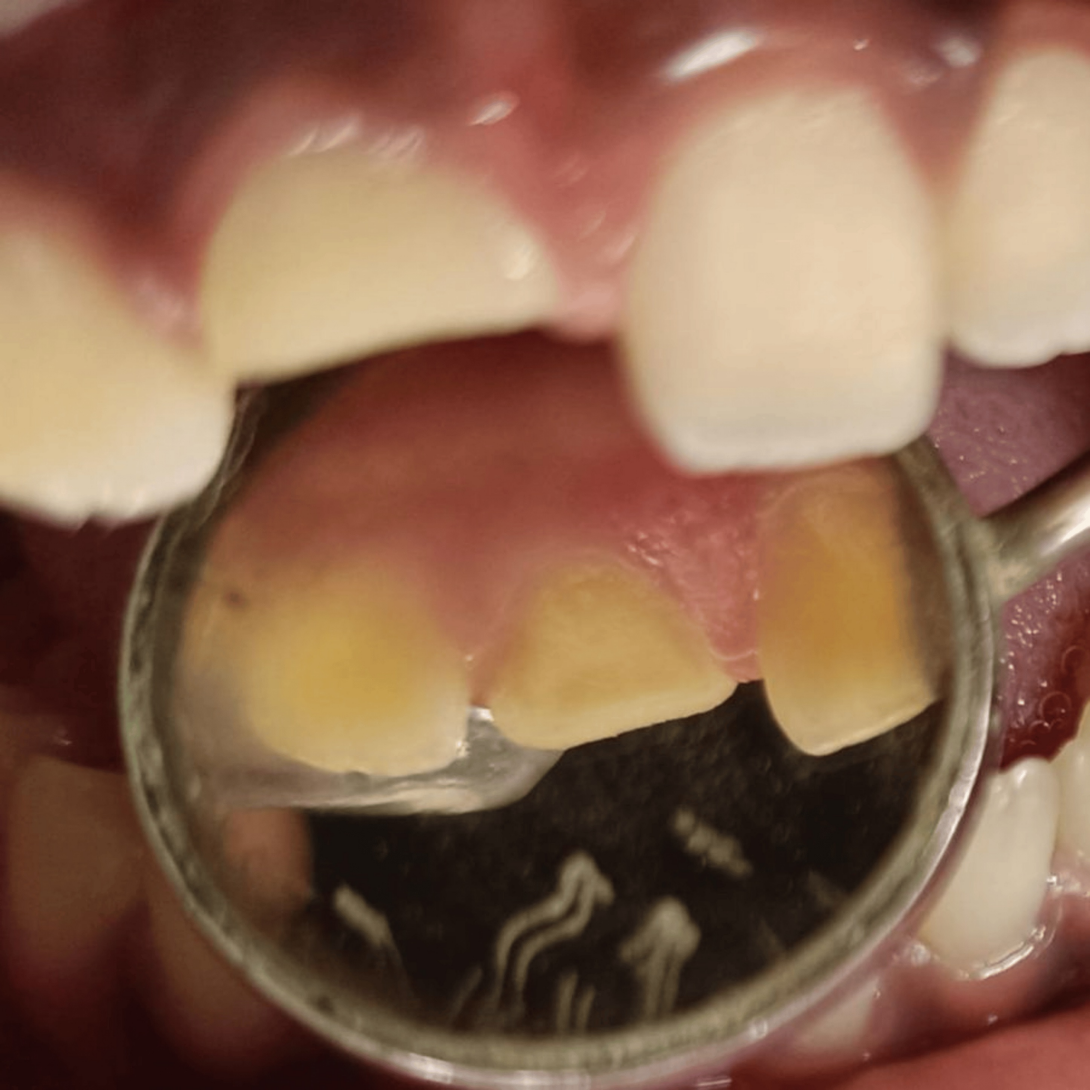
Clinical image showing scalloped bevel preparation along the fracture line of the maxillary right central incisor (tooth #11). The scalloped bevel design increases enamel surface area for bonding, enhances mechanical retention, and improves esthetic integration prior to fragment re‑attachment.

**Figure 4:**
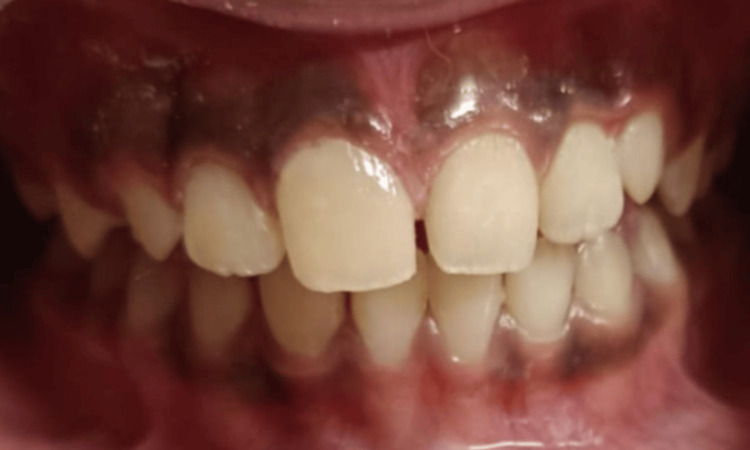
Post‑operative intraoral photograph showing successful fragment re‑attachment of the maxillary right central incisor (tooth #11) using scalloped bevel preparation and adhesive protocol, with restoration of esthetics and function.

The tooth surface was cleaned, and isolation was achieved using a rubber dam. Prior to re-attachment, a scalloped bevel preparation was placed along the fracture line on both the fractured tooth and the fragment. Subsequently, both surfaces were etched with 37% phosphoric acid for 15 seconds, rinsed, and gently air-dried. A bonding agent was then applied using an applicator tip, followed by light curing according to the manufacturer's instructions. The fragment was subsequently repositioned and re-attached (Figure 3). This scalloped bevel design increased the enamel surface area available for bonding, improved mechanical retention, and enhanced esthetic integration by allowing the composite resin to blend seamlessly with the natural tooth structure. Flowable composite resin was applied to the tooth surface, and the fragment was accurately repositioned. Excess resin was removed, and curing was performed from multiple directions to ensure complete polymerization (Figure 4). Finishing and polishing were carried out to restore esthetics and function.

The patient was advised to avoid biting hard foods and maintain good oral hygiene. Follow-up was taken at one week, one month, and three months, which revealed intact fragment retention, satisfactory esthetics, and absence of pulpal pathology.

## Discussion

Fragment re‑attachment has become a well‑accepted conservative treatment modality for managing uncomplicated crown fractures, particularly in children and adolescents. The technique preserves natural tooth tissue, restores esthetics, and provides psychological reassurance to patients and parents. Simonsen first introduced enamel beveling in 1979, recommending a 45° circumferential bevel on both the fragment and tooth margins to optimize etching and bonding [11]. This preparation removes minimal enamel and exposes enamel prisms in an end‑to‑end relationship, thereby enhancing micromechanical retention. The bevel preparation and simple re‑attachment showed similar retention rates [12]. Although Simonsen initially suggested that beveling concealed the fracture line beneath composite, he subsequently proposed an internal enamel groove to overcome discoloration issues associated with the labial composite band. A limitation of beveling is that preparation prior to re‑attachment may compromise the precise fit of the fragment. In the present case, a lotus petal bevel was employed, which provided increased bonding surface area, improved stress distribution, and superior esthetic blending compared to straight bevels.

The longevity of fragment re‑attachment is influenced by multiple factors, including the re‑attachment technique, adhesive system, fragment hydration, and use of intermediate materials [13-20]. Studies have shown that total‑etch adhesives provide higher fracture strength compared to self‑etch systems [21-24]. Intermediate materials such as glass ionomer cement, resin cements, and flowable composites have been tested, though their impact on fracture resistance remains inconclusive [25-28]. Garg et al. reported superior outcomes with flowable composites compared to resin cements [29,30]. While no method can fully replicate the strength of an intact tooth, appropriate adhesive protocols and preparation designs can achieve clinically acceptable strength and esthetics.

The condition of the fragment prior to re‑attachment is also critical. Farik et al. demonstrated that desiccation for more than one hour significantly reduces bond strength, recommending storage in hydrated media for at least 24 hours [31]. Prabhakar et al. showed that even 30 minutes of hydration can restore fracture resistance. Various storage media have been studied, including milk, coconut water, egg white, and Hanks’ Balanced Salt Solution. Among these, milk and tender coconut water consistently yield the highest fracture resistance values [32-35]. In the present case, the fragment was stored in saline, which maintained hydration and facilitated successful re‑attachment.

Advances in adhesive dentistry have enabled clinicians to adopt minimally invasive approaches such as fragment re‑attachment. The lotus petal bevel preparation used in this case enhanced bonding and esthetic outcomes, while proper fragment storage ensured optimal hydration. Although fracture resistance of re‑attached teeth may not equal that of intact teeth, the technique remains a reliable, cost‑effective, and esthetically superior option for managing traumatic crown fractures in pediatric patients.

## Conclusions

Fragment re‑attachment represents a conservative, esthetic, and cost‑effective treatment option for managing traumatic crown fractures in pediatric patients. The technique preserves natural tooth structure, restores function, and provides immediate psychological reassurance to both the child and parents. Although no preparation design or adhesive protocol can fully replicate the fracture resistance of an intact tooth, refinements such as scalloped bevels enhance bonding surface area, improve stress distribution, and achieve superior esthetic integration. The choice of adhesive system, intermediate material, and storage medium for the fragment plays a critical role in determining the long-term success of re‑attachment. In the present case, careful hydration of the fragment and the use of a lotus petal bevel preparation contributed to a favorable outcome, with intact retention, satisfactory esthetics, and absence of pulp pathology during follow‑up. With continued advances in adhesive dentistry, fragment re‑attachment remains a reliable and minimally invasive approach that should be considered the treatment of choice whenever the fractured fragment is available and clinically suitable.

## References

[1] Jalannavar P, Tavargeri A (2018). Influence of storage media and duration of fragment in the media on the bond strength of the reattached tooth fragment. Int J Clin Pediatr Dent.

[2] Pusman E, Cehreli ZC, Altay N, Unver B, Saracbasi O, Ozgun G (2010). Fracture resistance of tooth fragment reattachment: effects of different preparation techniques and adhesive materials. Dent Traumatol.

[3] Bourguignon C, Cohenca N, Lauridsen E (2020). International Association of Dental Traumatology guidelines for the management of traumatic dental injuries: 1. fractures and luxations. Dent Traumatol.

[4] Mahapatra J, Nikhade P (2021). Cosmetic enhancement of maxillary central incisors using rigid matrix technique. J Res Med Dent Sci.

[5] Baratieri LN, Monteiro S Jr, Caldeira de Andrada MA (1990). Tooth fracture reattachment: case reports. Quintessence Int.

[6] Lo Giudice G, Lipari F, Lizio A, Cervino G, Cicciù M (2012). Tooth fragment reattachment technique on a pluri traumatized tooth. J Conserv Dent.

[7] Cem Güngör H, Uysal S, Altay N (2007). A retrospective evaluation of crown-fractured permanent teeth treated in a pediatric dentistry clinic. Dent Traumatol.

[8] Garcia FC, Poubel DL, Almeida JC, Toledo IP, Poi WR, Guerra EN, Rezende LV (2018). Tooth fragment reattachment techniques-a systematic review. Dent Traumatol.

[9] Joshi A, Marwah N, Bandiwar M, Patni H, Singh V (2023). Biologic approach for fragment reattachment: a case report. J Dent Child.

[10] Simonsen RJ (1982). Restoration of a fractured central incisor using original tooth fragment. J Am Dent Assoc.

[11] Simonsen RJ (1979). Traumatic fracture restoration: an alternative use of the acid etch technique. Quintessence Int Dent Dig.

[12] Dean JA, Avery DR, Swartz ML (1986). Attachment of anterior tooth fragments. Pediatr Dent.

[13] Reis A, Francci C, Loguercio AD, Carrilho MR, Rodriques Filho LE (2001). Re-attachment of anterior fractured teeth: fracture strength using different techniques. Oper Dent.

[14] Rajurkar A, Chandak M, Nikhade P, Patel A, Taheri A, Bhongade S (2020). Evaluation of the bond strength of reattached incisal fragments using different techniques: an in vitro study. J Datta Meghe Inst Med Sci Univ.

[15] Diangelis AJ, Jungbluth M (1992). Reattaching fractured tooth segments: an esthetic alternative. J Am Dent Assoc.

[16] Kulkarni VK, Gadhe DE, Gavade SS, Dugad S, Khavnekar SS, Karpe HB (2022). Finite element analysis for fracture resistance of reattached human tooth fragment with different types of retentive preparation techniques. J Clin Pediatr Dent.

[17] Davis MJ, Roth J, Levi M (1983). Marginal integrity of adhesive fracture restorations: chamfer versus bevel. Quintessence Int Dent Dig.

[18] Abdulkhayum A, Munjal S, Babaji P (2014). In-vitro evaluation of fracture strength recovery of reattached anterior fractured tooth fragment using different re-attachment techniques. J Clin Diagn Res.

[19] Stellini E, Stomaci D, Stomaci M, Petrone N, Favero L (2008). Fracture strength of tooth fragment reattachments with postpone bevel and overcontour reconstruction. Dent Traumatol.

[20] Fatima S, Alam S, Kumar A, Andrabi SM, Rehman A (2021). Minimal intervention treatment of crown-root fracture in a mature permanent tooth by MTA pulpotomy and fragment reattachment: a case report. Aust Endod J.

[21] Karre D, Duddu MK, Swathi SS, Bin Mohsin AH, Bharadwaj B, Barshaik S (2018). Conservative vertical groove technique for tooth rehabilitation: 3-year follow-up. Case Rep Dent.

[22] Beltagy TM (2018). Laboratory and clinical evaluation of uncomplicated fragment reattachment using pinholes. Tanta Dent J.

[23] Walker M (1996). Fractured-tooth fragment reattachment. Gen Dent.

[24] Arhun N, Ungor M (2007). Re-attachment of a fractured tooth: a case report. Dent Traumatol.

[25] Demarco FF, Fay RM, Pinzon LM, Powers JM (2004). Fracture resistance of re-attached coronal fragments--influence of different adhesive materials and bevel preparation. Dent Traumatol.

[26] Brasil Maia G, Pereira RV, Poubel DL, Almeida JC, Dias Ribeiro AP, Rezende LV, Garcia FC (2020). Reattachment of fractured teeth using a multimode adhesive: effect of different rewetting solutions and immersion time. Dent Traumatol.

[27] Rajput A, Ataide I, Lambor R, Monteiro J, Tar M, Wadhawan N (2010). In vitro study comparing fracture strength recovery of teeth restored with three esthetic bonding materials using different techniques. Eur J Esthet Dent.

[28] Bhargava M, Pandit IK, Srivastava N, Gugnani N, Gupta M (2010). An evaluation of various materials and tooth preparation designs used for reattachment of fractured incisors. Dent Traumatol.

[29] Farik B, Munksgaard EC, Kreiborg S, Andreasen JO (1998). Adhesive bonding of fragmented anterior teeth. Endod Dent Traumatol.

[30] Garg A, Gupta S, Tewari N, Srivastav S, Chanda A (2023). Effect of adhesive materials in re-attachment of crown and crown-root fractures of permanent maxillary anterior tooth: a computational study. Math Comput Appl.

[31] Farik B, Munksgaard EC, Andreasen JO, Kreiborg S (1999). Drying and rewetting anterior crown fragments prior to bonding. Endod Dent Traumatol.

[32] Prabhakar AR, Yavagal CM, Limaye NS, Nadig B (2016). Effect of storage media on fracture resistance of reattached tooth fragments using G-aenial Universal Flo. J Conserv Dent.

[33] Hegde RJ, Kale SJ. (2017). Comparison of the effect of various storage media on the fracture resistance of the reattached incisor tooth fragments: an in vitro study. Indian J Dent Sci.

[34] Trivedi S, Bansal A, Kukreja N (2022). Evaluation of fracture resistance of reattached fractured tooth fragment stored in different storage media: an in vitro study. J Contemp Dent Pract.

[35] Rathod P, Mankar N, Nikhade P, Chandak M, Patel A, Ikhar A (2024). Reattachment of fractured tooth: a comprehensive review. Cureus.

